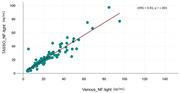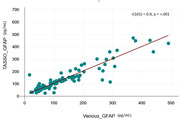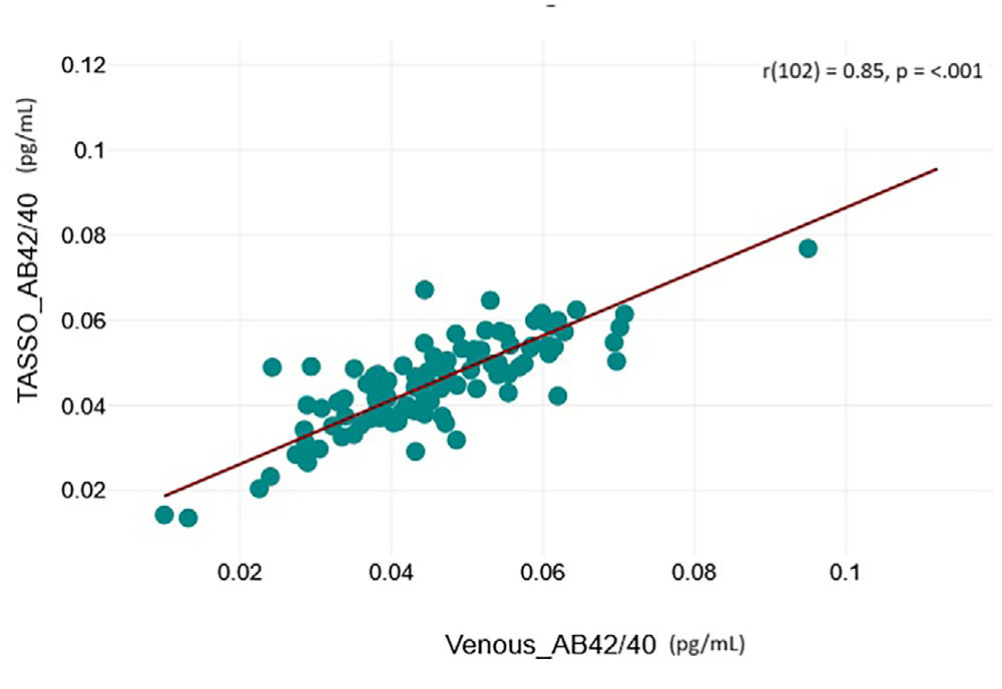# Performance of 2 Point‐of‐Care Capillary Plasma Collection Devices for Alzheimer’s Disease Biomarker Testing

**DOI:** 10.1002/alz.095589

**Published:** 2025-01-09

**Authors:** Sara G Ho, Alexandria Lewis, Megha Patel, Anny Zheng, Abhay Moghekar

**Affiliations:** ^1^ Johns Hopkins University School of Medicine, Baltimore, MD USA

## Abstract

**Background:**

As blood‐based biomarkers become critical to Alzheimer’s Disease (AD) clinical testing, establishment of less invasive plasma collection methods are key. We compared the Tasso+ and the TAPII capillary whole blood collection devices to traditional venipuncture for safety and efficacy in our clinic and measured AD biomarkers neurofilament‐light (NFL), glial fibrillary acidic protein (GFAP), phosphorylated‐tau 217 (ptau217), and amyloid‐beta 42/40 ratio (Aβ42/40) in cerebrospinal fluid (CSF), capillary, and venous plasma to assess potential age and cognition related group differences.

**Method:**

Patients seen at the Johns Hopkins Center for CSF Disorders (N = 193) underwent diagnostic lumbar punctures and/or extended lumbar drainage procedures. After CSF and venous plasma was collected, warming packs were held to the patient’s upper arm site for 5 minutes before capillary blood was collected via TAPII or Tasso+ device for 5 minutes. Post processing, CSF, venous, and capillary plasma was analyzed using Quanterix SIMOA immunoassays. Clinical Dementia Ratings and Montreal Cognitive Assessment scores were also collected.

**Result:**

86 TAPII and 121 Tasso+ plasma samples were collected from patients (78 M, 115 F) with mean age 61 years ± 18.3 (range 16‐91 years). The mean volume for Tasso+ samples was 294.1uL±127.2 and 266.94uL±18.71 for TAPII though this difference was not statistically significant. Tasso+ devices were 90.3% successful in collecting >50uL whole blood, and TAPII devices were 86% successful. NFL, GFAP, and Aβ42/40 ratio in CSF demonstrated moderate positive correlations with both venous and capillary plasma, although TASSO+ samples showed stronger correlation with venous plasma than TAPII (NFL Tasso r = 0.93, p<0.001, TAP r = 0.85, p<0.001; GFAP Tasso r = 0.9, p<0.001, TAP r = 0.85, p<0.001; Aβ42/40 Tasso r = 0.72, p<0.001, TAP r = 0.86, p<0.001). Ptau217 CSF measurements showed a weaker positive correlation with venous and capillary samples (Venous r = 0.34, p<0.001, Tasso r = 0.19, p = 0.048, TAP 0.05, p = 0.623).

**Conclusion:**

Neurodegenerative biomarkers measured in capillary plasma as collected by the Tasso+ and TAPII correlated well with similar measures obtained from venous plasma. Tasso+ and TAPII devices performed similarly regarding success rate and volume collected.